# Continuous Norming Approaches: A Systematic Review and Real Data Example

**DOI:** 10.1177/10731911241260545

**Published:** 2024-07-27

**Authors:** Julian Urban, Vsevolod Scherrer, Anja Strobel, Franzis Preckel

**Affiliations:** 1 University of Trier, Germany; 2 GESIS —Leibniz Institute for the Social Sciences, Mannheim, Germany; 3 University of Technology, Chemnitz, Germany

**Keywords:** continuous norming, systematic review, regression-based norming, norm generation, need for cognition, cNORM, GAMLSS

## Abstract

Norming of psychological tests is decisive for test score interpretation. However, conventional norming based on subgroups results either in biases or require very large samples to gather precise norms. Continuous norming methods, namely inferential, semi-parametric, and (simplified) parametric norming, propose to solve those issues. This article provides a systematic review of continuous norming. The review includes 121 publications with overall 189 studies. The main findings indicate that most studies used simplified parametric norming, not all studies considered essential distributional assumptions, and the evidence comparing different norming methods is inconclusive. In a real data example, using the standardization sample of the Need for Cognition-KIDS scale, we compared the precision of conventional, semi-parametric, and parametric norms. A hierarchy in terms of precision emerged with conventional norms being least precise, followed by semi-parametric norms, and parametric norms being most precise. We discuss these findings by comparing our findings and methods to previous studies.

## Introduction

Gaining six IQ points just by aging a month? While such a gain may be individually desirable, it must clearly be a methodological artifact and not a real increase in intelligence (Zachary & Gorsuch, 1985). More precisely, it can be explained by conventional norming, the most applied method in psychology to interpret individual test results (Urbina, 2014, p. 83). Conventional norming can result in biased norms when there are too few and/or too small subgroups for test score standardization (Timmerman et al., 2021). Biased norms are of concern for important test-based decisions such as college entrance (Duncan et al., 2011), selection in assessment centers, or clinical diagnosis (Brandt & Moosbrugger, 2020). Continuous norming methods offer some solutions to biased norms. In this article, we conduct a systematic review of current research on continuous norming methods. In addition, we address some open questions identified in our review using a real data example. Our findings shed light on (a) current practices in continuous norming, (b) the effect of the norming method on norm precision, and (c) on individual test scores.

### Conventional Norming and Related Problems

To interpret test scores, conventional norms express the individual test score in a standardized way with respect to a specific reference population (American Educational Research Association et al., 2014, p. 96; Urbina, 2014, p. 78). This reference population is usually defined by sociodemographic variables such as age, hereafter referred to as norm predictors. For example, an individual’s reference population may be the population of a country between the ages 16 and 18. While raw scores lack comparability, the standardized scores control for the norm predictors and are therefore independent of the norm predictors.

To standardize an individual raw score, norming compares it to the typical scores of a reference group with similar norm predictor characteristics (Urbina, 2014, p. 83). This requires differentiated norms for different levels of the norm predictor as well as the representativeness of the respective subgroup data. Conventional norming achieves this norm differentiation by computing norms for subgroups of the norm predictor (Urbina, 2014, p. 83). Such so-called within-subgroup norms are straightforward for categorical norm predictors such as school type but become arbitrary for continuous variables such as age. Traditionally, test developers divide the continuous norm predictor into intervals and compute norms within each subgroup using linear transformation, percentiles, or area transformation. These three methods have no specific data assumptions. Linear transformation uses the mean and standard deviation of each subgroup for linearly transforming the raw score to any scale (e.g., z-scores or T-scores). Linear transformation preserves the raw score distribution within each subgroup. Percentiles express a person’s rank within a subgroup. Area transformation uses this rank within a subgroup and then inverse normal transformation to extract a normalized z-score, which is the norm score (A. Lenhard et al., 2018a).

**Figure 1.**
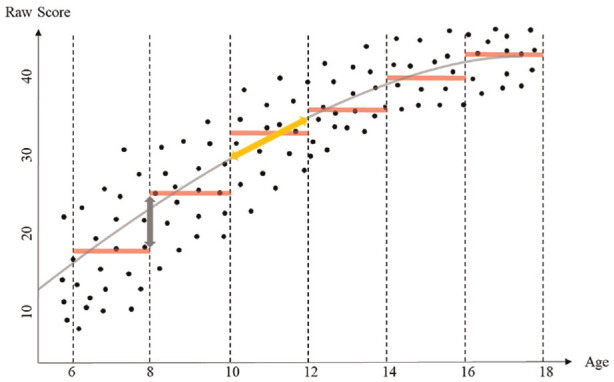
Association of Norm Predictor and Fictional Raw Scores. *Note*. Dashed lines indicate boundaries of subgroups. Red lines show the median for these subgroups. The orange arrow refers to within-subgroup bias, the gray arrow to transition bias. The blue error displays the rationale of our within-subgroup bias proxy. The gray curve displays a continuous relationship of raw scores and norm predictor age.

Conventional norming methods often result in biased norms (Timmerman et al., 2021), meaning that norm scores are systematically over- or underestimated for some individuals. To illustrate the origins of bias, Figure 1 depicts the association between fictional test raw scores and the norm predictor age. The dashed vertical lines represent the age subgroup boundaries, and the red horizontal lines show the subgroup medians. The double-headed arrows indicate two sources of bias. The orange arrow indicates within-subgroup bias. Individuals at the margins of a subgroup are systematically over- or underestimated because there is still an effect of the norm predictor within the subgroup. Thus, a remaining correlation between norm scores and norm predictor within a subgroup introduces bias. The gray arrow indicates the transition bias. The same raw score can lead to way different norm scores solely because an individual is compared with different age subgroups. This bias is especially pronounced for individuals whose age is close to the transition point (e.g., if the same individuals are tested with a month difference and the transition point for switching to the norms of the next age subgroup falls within this period). Transition bias increases with larger median differences between the transitioned groups.

Importantly, both biases increase as the (age) subgroup width increases. Narrowing the width of age subgroups is a potential solution to reduce bias. However, this poses a precision challenge on norming unless the subsample size, and thus, the overall sample size is increased. Without increasing the overall sample size, narrower age subgroups result in smaller sample sizes per subgroup. Smaller sample sizes increase the likelihood of randomly oversampling individuals with a particular score level. Consequently, the uncertainty of the association between the raw score and the norm score, the sampling error grows, while the inverse of the sampling error, the precision, decreases (e.g., Crompvoets et al., 2021; W. Lenhard & Lenhard, 2021). To achieve sufficient precision, sample size guidelines for conventional norms recommend a minimum of *n* = 400 per subgroup (Eid & Schmidt, 2014, p. 381). However, such guidelines are not generally applicable as they depend on, for example, the domain of application and the norming population (Evers et al., 2013). Moreover, the collection of normative data for narrow age subgroups is no longer feasible following these guidelines. This leads to an economy-precision dilemma.

### Continuous Norming

Continuous norming methods offer a solution to the economy-precision dilemma. These methods use the entire sample to compute norm scores. As an example, the gray curve in Figure 1 shows the continuously modeled median. Since the first article by Zachary and Gorsuch (1985), various methods have emerged, namely inferential norming (e.g., Zhu & Chen, 2011), semi-parametric norming (e.g., A. Lenhard et al., 2018a), and (simplified) parametric norming (e.g., Timmerman et al., 2021; Van Breukelen & Vlaeyen, 2005).

#### Inferential Norming

Inferential norming involves regressing distributional parameters (such as mean, standard deviation, skewness, and kurtosis) of raw scores on discretized norm predictor values using linear or polynomial regression (Zachary & Gorsuch, 1985; Zhu & Chen, 2011). The resulting regression equation allows the computation of distribution parameters for each possible value of the norm predictor. With these specific parameters, it is possible to transform raw scores into norm scores, for example, by linear transformation. The resulting norm scores can be hand smoothed (e.g., Zhu & Chen, 2011). If no other distribution parameters than central tendency are considered, the assumption for this modeling is homoscedasticity and normality of the residuals.

#### Semi-Parametric Norming

Semi-parametric norming combines conventional norms with regression models. After computing conventional norms via area transformation, the raw score is regressed on these norm scores as well as the continuous norm predictor using stepwise polynomial regression (A. Lenhard et al., 2018a). This stepwise regression varies the power of these polynomials from zero to a specified maximum level (e.g., four) and uses all possible product terms (interactions) as predictors. The significant terms of this regression form a Taylor-Polynomial, which is sufficient to compute norm scores as a function of raw score and norm predictors. This method does not assume a specific data distribution but requires careful model selection to avoid both under- and overfit (Gary et al., 2021), as such a misfit can lead to biased norms.

#### (Simplified) Parametric Norming

Parametric norming methods apply regression models of the *Generalized Additive Models for Location, Scale, and Shape* (GAMLSS; Rigby & Stasinopoulos, 2005). GAMLSS include a plethora of probability density functions (PDFs) to model the raw score distribution. Consequently, GAMLSS users must choose a specific PDF with its associated distribution parameters, as well as a linear or smooth function to relate the norm predictor to the distribution parameters. The choice of a smooth function allows the association between the norm predictor(s) and these distribution parameters to be modeled in a non-linear way. For example, the gray curve in Figure 1 could be the result of a GAMLSS regression assuming a Poisson distribution with power polynomials as smooth function. After these initial decisions, statistical modeling, fit evaluation, and model selection take place (Timmerman et al., 2021). With the final model, continuous norm scores can be computed. Although parametric norms do not require groups of the continuous norm predictor, they do depend on the PDF chosen. Each PDF makes certain assumptions about the data. Thus, model selection needs to consider data characteristics and balance under- and overfitting (Voncken et al., 2021a).

Some researchers use a simplified version of parametric norming, which is also known as regression-based norming (e.g., Crompvoets et al., 2021; Van Breukelen & Vlaeyen, 2005). Since all continuous norming methods are regression-based, we refer to this method as simplified parametric norming. In simplified parametric norming the raw scores are regressed on norm predictors using linear or more complex terms. This assumes normally distributed residuals and homoscedasticity. Violations of this assumption can result in biased norm scores (Crompvoets et al., 2021). GAMLSS includes the normal distribution. However, researchers using these simplified parametric norming models do not regularly perform a thorough PDF selection and fit evaluation.

In summary, continuous norms reduce within-subgroup and transition bias but may themselves be biased if model assumptions are not met. The four methods differ in their assumptions and complexity. A recent overview of German tests (Urban et al., in press) found that conventional norms are commonly used, but that there is a small trend toward continuous norms. Such scarce use may indicate a lack of clear guidelines regarding the norming procedure of psychometric tests and scales. In fact, there is no finite consensus or guidelines on when to use which continuous norming method. To add to our knowledge in this field, we (a) provide a systematic review of the continuous norming literature and (b) address some of the research gaps identified in this review in a real data study.

## Systematic Review of Continuous Norming Literature

We conducted a systematic literature review following PRISMA guidelines (Page et al., 2021). We aimed to (a) summarize previous research on continuous norming, (b) derive the exact applied continuous norming method, (c) examine the characteristics of each norming sample, and (d) compare different continuous norming methods.

### Method

#### Search Strategy & Eligibility Criteria

We conducted a standardized literature search in November 2022 using the databases PsycINFO, Web of Science, and ERIC. Our final search string included the following terms: “continuous norming,” “semi-parametric norming,” “parametric norming,” “regression-based norming,” “regression-based normative data,” “cNORM,” “GAMLSS AND norming,” as well as “inferential norming.” We searched all fields in the mentioned databases and did not apply any filters. This search resulted in 281 studies. We augmented our search with a forward search of papers introducing a new norming method (e.g., A. Lenhard et al., 2018a; Timmerman et al., 2021; Zhu & Chen, 2011) and a backward search in all included papers.

A study had to meet four criteria to be included in the review. First, it had to conduct or report on the process of any form of continuous norming. While this included test reviews published as journal articles, it led to the exclusion of studies that conducted research with norm data (e.g., Breit et al., 2020). Second, raw scores should neither reflect a change score, nor should norm scores reflect a (multi-)nominal classification scores (e.g., Hessler et al., 2017). Change scores use a different input (change scores instead of test raw scores), and classification scores form a different output (multinominal instead of continuous). Third, we only included peer-reviewed journal articles and dissertations. Fourth, the language of the article or dissertation had to be English or German.

#### Study Selection and Data Coding

**Figure 2.**
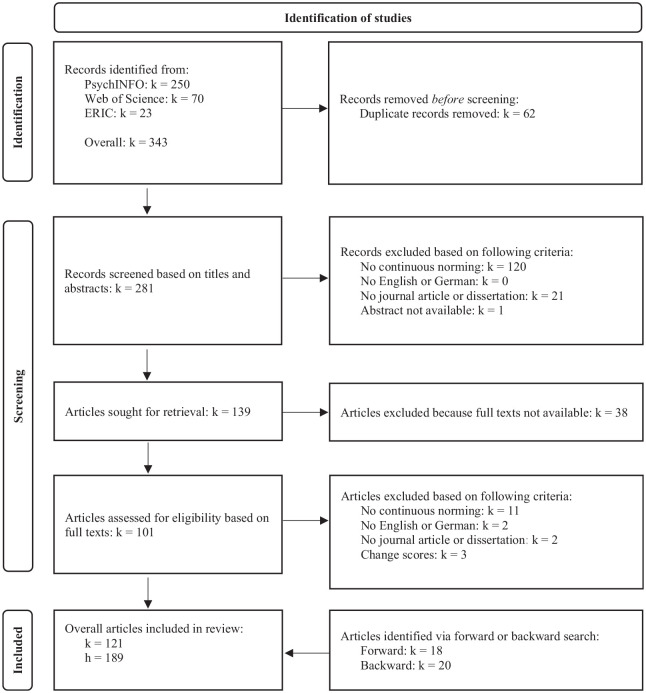
Study Identification and Screening Process as a PRISMA Chart. *Note.* This figure presents the literature search and gives information on the number of coded studies. *k* = number of publications, *h* = number of included studies.

Figure 2 shows a detailed overview of the selection process including the reasons for exclusion. The first author screened all 281 abstracts. The second author independently screened 50 randomly selected abstracts. Of the 139 retained publications, 101 full texts were accessible. Of those, we included 83 publications. The forward search of methodological papers (e.g., A. Lenhard et al., 2018a; Timmerman et al., 2021; Zhu & Chen, 2011) led to the inclusion of 18 further publications. The backward search led to 20 further publications. Thus, we included 121 publications from which we coded a total of 189 studies.

The coded data items were clustered into five areas. First, the *norming method* indicated all norming methods used by a study. We distinguished between conventional norms (linear or area transformation), continuous norms (inferential, semi-parametric, [simplified] parametric norming), or other methods (e.g., Bayesian regression). Second, *sample characteristics* described overall sample size, norm predictor range, as well as subsample size and subsample width for conventional norms. Third, *data type* indicated whether a study used real and/or simulated data. Fourth, we coded the normed *tests* and their *psychological area* (e.g., intelligence, personality, and neuropsychology). We identified the area by the keywords of the study, the construct definition of the test, or other studies using the same test. Finally, *study aim* differentiated whether a study computed norms, compared norming methods, or had other objectives.

We were not able to extract all variables of interest for all studies. We coded values as missing if there was uncertainty (e.g., the article did not state whether they performed conventional norming via area or linear transformation). We coded a study multiple times if it included several tests or both data types. This led to 189 coded studies reported in 121 publications.

#### Bias Assessment and Synthesis Method

We determined the inter-rater reliability to assess the agreement between the two coders in study inclusion (Krippendorff, 1980, p. 134). We synthesized the data descriptively. Each case with valid data for a given variable was eligible for the respective analysis. Of interest were the number of cases using a specific method, norm sample characteristics, and the psychological area of the real data studies. Moreover, we summarized the main findings qualitatively, particularly for studies comparing different norming methods.

### Results

The codesheet, the PRISMA-checklist, references of the included studies, and the used R-Code are available as Supplemental material (https://osf.io/g94ey/; ESM 1–4).

There were discrepancies in the coding for five of the 50 abstracts, resulting in a Krippendorff’s alpha of α = .76. All discrepancies consisted of the first author including a study that the second author excluded. We discussed the reasons for the discrepancies. The first author had a more liberal inclusion criterion regarding explicit mention of continuous norming in the abstract. To ensure sensitivity of our study inclusion, we screened all full texts included by the first author. Of the five discrepant studies, four met our inclusion criteria.

#### Study Characteristics

**Figure 3.**
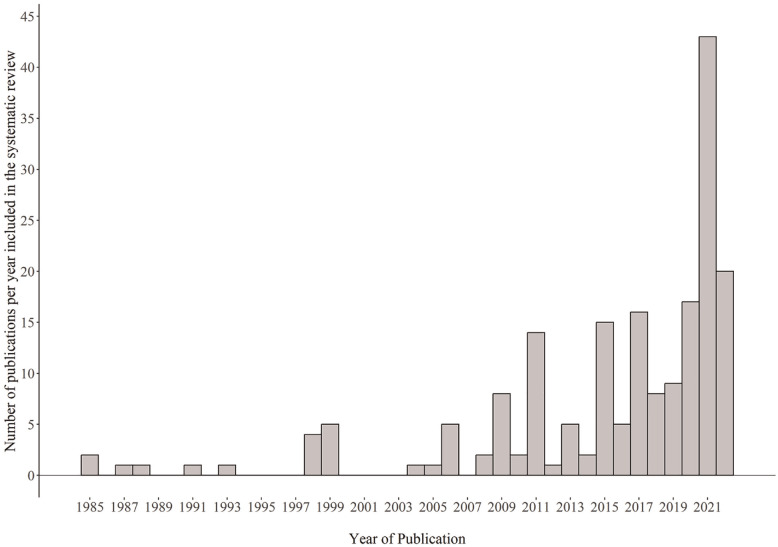
Number of Publications per Year. *Note.* Number of cases *N* = 189.

Table 1 summarizes sample characteristics and year of publication. The included tests cover a broad range of psychological areas (e.g., development or achievement) and a wide age range (0.83–102 years). Half of the studies included only adults (*Mdn_lower age_* = 18). Studies that computed conventional norms in addition to continuous norms typically had a small subgroup sample size (*Mdn* = 50) and age intervals larger than 1 year (*Mdn* = 14 months). The first paper on continuous norming was published in 1985. Nonetheless, the majority of papers have been published more recently (*Mdn* = 2018). Figure 3 presents the number of papers published per year. Table 2 summarizes the data type, study aim, and psychological area. Most studies used real data and computed norms for a neuropsychological test.

**Table 1:** Mean Values, Standard Deviations, Median, Minimum, Maximum, and Respective Number of Cases for Publication Year, Norm Sample Size, Age Boundaries in Norm Sample, Sample Size per Subgroup, Subgroup Width, and Age Range in the Norm Sample.

Variable/statistic	*n*	*M*	*SD*	*Mdn*	*Min*	*Max*
Publication year	189	2015	7.77	2018	1985	2022
(min) *N*	184	1,460.0	2,159.09	500	55	12,350
Lower age [years]	175	27.0	18.90	18	0.83	62
Upper age [years]	175	71.6	27.91	82	2.5	102
Number of groups	6	6.5	1.38	6	5	9
(min) *n* per group	5	54.4	50.76	50	3	112
Interval [months]	8	50.8	54.23	14	6	120
Range age [years]	175	44.6	23.07	45	1.67	82

*Note.* Values for number of groups, (min) *n* per group, and interval refer to conventional norms also reported in included studies.

**Table 2:** Study Characteristics of Included Cases.

Variable/statistic	Real data	Simulated data	Total
Study aim			
Computing Norms	178	6	182
Comparing Norming Methods	4	3	7
Other	2	2	4
Norming method			
Conventional Norming	9	1	10
Area Transformation	3	0	3
Linear Transformation	3	0	3
Inferential Norming	12	0	12
Semi-parametric Norming	6	3	9
Simplified Parametric Norming	148	3	151
Parametric Norming	4	5	9
Other	9	2	11
Psychological area			
Neuropsychological	132	-	132
Intelligence	18	-	18
Clinical	17	-	17
Academic Achievement	5	-	5
Development	4	-	4
Memory	3	-	3
Total	179	11	189

*Note*. Some studies used both data types and had multiple aims.

#### Continuous Norming Method

Most studies applied simplified parametric norming (*n* = 151). Seventy-four of these studies used multiple regression with linear effects of the norm predictor, 68 studies used more complex terms such as quadratic, higher power polynomial, logarithmic, square root, or inverse terms. Only four studies used multilevel regression. Seventy-three studies tested the assumptions of simplified parametric norming such as no multicollinearity, homoscedasticity, or normality (e.g., Lorentzen et al., 2021; Testa et al., 2009). Moreover, 36 studies modeled linear effects across large age intervals (>45 years; for example, Dassanayake & Ariyasinghe, 2019; Tallberg et al., 2008) without reporting tests for linearity.

Overall, 12 studies applied inferential norming. Ten of these studies computed norms for real data tests. Two studies are test reviews published as journal articles. All studies included non-linear effects of the norm predictor on the raw scores. However, only two of the studies computing norms modeled the skewness as distributional parameter in addition to the mean and the standard deviation (Selvam et al., 2016; Zhu & Chen, 2011). The two test reviews describe that the norming procedure accounted for skewness (Cormier et al., 2016) and skewness and kurtosis (Schmitt & Decker, 2009).

Nine studies investigated semi-parametric norming and were published after 2017. Here, norms exist for five real data tests. One study compared semi-parametric with conventional norms and two studies compared them with parametric norms. Eight studies reported information on modeling and fit evaluation. Articles by the developers of semi-parametric norming (A. Lenhard et al., 2018a) reported the most detailed information; other authors described their models as supplements (Echemendia et al., 2021) or did not report clear information on the model (Rossi et al., 2022).

Overall, nine studies used parametric norms. Three studies computed norms for real data tests. These three real data studies compared the fit of different models. All nine studies differed in their assumed PDFs and modeled smooth functions. The final GAMLSS models utilized either a normal, a (truncated) Box-Cox-Power-Exponential (BCPE), a Box-Cox (BC), a Beta-Binomial (BB), a skew student’s *t*, or a Sinushyperbolicus-Arcussinushyperbolicus (ShaSh) distribution as PDF. Smooth functions were Basis-splines, Panelized-splines (P-splines), or power polynomials respectively. Although GAMLSS models can vary in PDF and smooth functions, all studies except the Timmerman et al. (2021) tutorial fixed either the smooth function (Gary et al., 2021; A. Lenhard et al., 2019; Vugteveen et al., 2022) or the PDF (Voncken et al., 2019a, 2019b, 2021a). Such fixations hinder thorough model selection.

Eleven cases applied other methods for continuous norming, computing norms for eight real test data. The association between the raw score and norm predictor was modeled by fractional polynomials (Casaletto et al., 2015; Krishnan et al., 2004; Norman et al., 2011), quantile regression (Crompvoets et al., 2021), or Bayesian regression (Voncken et al., 2021b; Wang et al., 2020).

#### Findings for Comparisons of Continuous Norming Methods

Seven publications with overall eight studies compared different norming methods: six studies compared conventional norms with inferential (*n* = 1), semi-parametric (*n* = 1), simplified parametric (*n* = 2), or other continuous norms (*n* = 2); two studies compared semi-parametric with parametric norms. Oosterhuis et al. (2016) also included the comparison of conventional and simplified parametric norms. We discuss this study later because of its focus on sample size requirements.

Zachary and Gorsuch (1985) found that inferential norming with linear and quadratic terms had an advantage over conventional norms in terms of transition bias and the mean of the norm data. They used a sample of overall *N* = 1,880 and each subgroup included at least *N* = 160 participants. Burggraaff et al. (2017) found that simplified parametric norms estimated with linear and quadratic terms had a higher sensitivity for classifying individuals as impaired in terms of their neuropsychological functioning. Their sample included 96 healthy controls and 157 patients. The study of Crompvoets et al. (2021) compared conventional norms with simplified parametric norms and quantile regression in multiple simulated samples. Simplified parametric norming and quantile regression were more precise than the conventional norming, but the estimated norm scores were biased. This bias occurred particularly for skewed data and in absence of homoscedasticity. Sample size did not influence bias and the precision hierarchy but affected absolute precision. Thompson et al. (2022) examined mean differences between conventional and continuous norms estimated with hierarchical linear modeling for a large sample *N* > 11,000. They found only negligible effects between conventional and continuous norm scores (all |*d*| ≤.04). A. Lenhard et al. (2018) compared conventional and semi-parametric norms. For a large sample (*N* > 3,500) and smaller cross-validation samples (*N* ~ 675) they found a higher precision of semi-parametric norms as well as independence of age subgroup width for semi-parametric norms.

Two studies compared semi-parametric with parametric norms (Gary & Lenhard, 2021; A. Lenhard et al., 2019). Both studies are simulation studies that varied sample size and scale difficulty. Gary and Lenhard (2021) also varied the number of items. The modeled GAMLSS models varied in the assumed PDF (normal distribution, BC, [truncated] BCPE, or ShaSh) but only used Basis-splines as smooth function. For easy and difficult scales, parametric norms were more biased than semi-parametric norms, as indicated by a stronger deviation of parametric norm scores from the population norm scores. The effect vanished for scales of medium difficulty and diminished with an increasing number of items except for the GAMLSS model assuming a normal distribution. Semi-parametric norms showed on average less bias for all modeled sample sizes, but bias decreased for both norming methods with an increasing sample size. However, these results are highly dependent on the study conditions. Different scale characteristics (i.e., skewness, number of items, response scales) and different modeling conditions for parametric norming (i.e., modeled PDF and smooth /linear function) may have yielded other results.

To conclude, the comparison studies suggest that continuous norms have a higher precision than conventional norms. Nonetheless, continuous norms may be biased if model assumptions are violated. Moreover, the results of the comparison studies may not generalize to other data sets or other simulated conditions. The exact circumstances under which a specific continuous norming method is preferable are yet unclear. Therefore, there is no definitive conclusion on when to use which continuous norming method.

#### Continuously Normed Tests

Overall, the 179 real data studies provide continuous norms for 108 different tests. These 108 tests were mostly neuropsychological (*n* = 72; 66.1%), followed by clinical (*n* = 15; 13.8%), intelligence (*n* = 12; 11%), development (*n* = 4; 3.67%), achievement (*n* = 3; 2.75%), and memory tests (*n* = 3; 2.75%). The Code sheet in the Supplemental materials (ESM 1) reports the tests.

#### Sample Size Requirements

Three studies examined sample size requirements for inferential norming (Zhu & Chen, 2011) or simplified parametric norming (Innocenti et al., 2023; Oosterhuis et al., 2016). Innocenti et al. (2023) provide formulas and tables for the required sample size. They discuss the issue of violated model assumptions and suggest solutions via segmented regression or GAMLSS PDFs with less strict assumptions. Nonetheless, their recommendations have limitations. Their formulas are based on predetermined normal models and require a minimum sample size to be unbiased. Moreover, the formulas neglect the relevance of the test reliability to estimate confidence intervals. Thus, the guidelines are useful if one has founded assumptions about the model, does not focus on individual diagnostics, and the sample size exceeds the minimum sample size required for the formulas to be unbiased.

Oosterhuis et al. (2016) compared the sample size requirements for conventional and simplified parametric norming. For the conditions modeled, simplified parametric norms had a higher precision and consequently required a smaller sample size than conventional norms to achieve the same precision. The study also provides information on the association of sample size and precision as a function of the construct level (i.e., percentile). The applicability of this guideline is limited. It is only valid for assumed linear effects of the norm predictor on the test scores, normally distributed residuals, and in the presence of homoscedasticity.

Zhu and Chen (2011) applied inferential norming with age subgroups within a given overall age range. They varied the sample size per group and, consequently, the overall sample size. Thus, effects of subsample size and overall sample size are not distinguishable. To summarize, no studies investigated sample size requirements for semi-parametric or parametric norming and the available sample size guidelines for inferential or simplified parametric norming are not generally applicable.

### Summary

Our review highlighted the increasing interest in and application of continuous norming methods. Most studies used simplified parametric norming (80%) and only a few used inferential norming (6%), semi-parametric norming (5%), or parametric norming (5%). There are some critical issues in norming practice such as small (subgroup-) samples over a very large age range or lack of testing essential model assumptions. Although continuous norms can address within-subgroup and transition bias, they can introduce bias if model assumptions are violated. Thus, evaluation of model assumptions and model fit are crucial to ensure unbiased and precise norms.

We identified three open questions in the existing literature. First, there is no finite conclusion regarding the preferred norming method. This includes the lack of real data studies comparing semi-parametric and parametric norms. Second, even continuously normed tests usually present classical norm tables in which a norm score is assigned to each raw score (e.g., Grob & Hagmann-von Arx, 2018; Kubinger & Holocher-Ertl, 2014; W. Lenhard et al., 2017). Studies investigating whether this discretization of the norm predictor may introduce transition or within-subgroup biases are rare. Third, the question of whether an individual norm score varies significantly as a function of the norming method is neglected. Only Thompson et al. (2022) investigated differences between individual conventional and continuous norm scores. They only tested for mean group differences, where positive and negative differences can compensate, wherefore the effect of the norming method on individual test scores remains unclear.

## Real Data Example

We addressed some of these open questions by comparing age-dependent conventional, semi-parametric and parametric norms. We used the original norm data from a personality questionnaire measuring *Need for Cognition* (NFC) in primary-school students (the NFC-KIDS; Preckel & Strobel, 2017).

First, we compared the precision of all three methods (i.e., age-dependent conventional, semi-parametric, and parametric norming). To ensure robust results, we used two different numbers of age subgroups for this comparison. This allowed us to control for subgroup sample size as an alternative explanation for our findings. However, the number of age subgroups should only affect conventional norming (A. Lenhard et al., 2018a). We expect a precision advantage for continuous norms over conventional norms, because previous studies have reported this advantage for continuous norms, regardless of sample size and data skewness (Crompvoets et al., 2021; A. Lenhard et al., 2018a; W. Lenhard & Lenhard, 2021). Some studies report a precision advantage of semi-parametric norming over parametric norming, especially for skewed data and a smaller number of items (Gary & Lenhard, 2021; A. Lenhard et al., 2019). However, these studies lack a thorough parametric model selection (Timmerman et al., 2021). If a thorough model selection is performed, we expect a higher precision for parametric norms compared with semi-parametric norms because parametric norms do not depend on the construction of sample-dependent conventional norms in a first step.

Second, we aimed to examine the question whether providing discretized norm tables for continuously normed tests introduces transition and within-subgroup bias. Presenting classical discretized norm tables showing the norm scores assigned to each raw score is quite common even for continuously normed tests (e.g., Grob & Hagmann-von Arx, 2018; Kubinger & Holocher-Ertl, 2014; W. Lenhard et al., 2017). Although this eases the usage for test applicants, it requires the building of subgroups. Subgrouping introduces the risk that a correlation between norm predictor and norm score may persist (i.e., within-subgroup bias). Moreover, an individual’s norm score may change significantly when compared with the neighboring subgroup despite having the same raw score (i.e., transition bias). The smaller the number of subgroups, the greater the risk of introducing transition and within-subgroup bias.

Third, we analyzed the differences between conventional and continuous norm scores at the individual level. We assessed the influence of the number of age subgroups on these differences. We expected larger differences between conventional and continuous norm scores with an increasing number of groups due to the decreasing precision of conventional norms.

Finally, we examined the ability of the resulting norm scores to reduce the correlation of the assessed trait with age. Norms express the raw scores in a standardized way, that is, independent of the norm predictor. Thus, the correlation of norm scores and age should be negligible.

### Method

#### Sample

We used data of the original NFC-KIDS (Preckel & Strobel, 2017) norming sample. Data collection took place between September 2012 and February 2014 in primary schools (students in Grades 1–4) across six federal states of Germany. Students were assessed in their classrooms. Overall, 2,763 children participated. We excluded 182 cases due to missing values on five or more NFC items (*n* = 30), on age (*n* = 151), or due to having an extreme age value (*n* = 1; the respective individual was 7 months younger than the rest of the sample). Thus, our analyses sample consisted of 2,581 students. These students ranged in age from 71 to 146 months (*M* = 107.08, *SD* = 15.18). Parents of 1,001 children (48.59%) reported a female sex and 1,059 parents (50.51%) reported a male sex for their child. All students had parental permission, participated anonymously, and could abort participation at any time without giving any reasons. For an extensive overview of the data collection and additional demographic information, see Preckel and Strobel (2017).

#### Instruments

*Need for Cognition* (NFC) is defined as an individual’s “tendency to engage in and enjoy thinking” (Cacioppo & Petty, 1982). The NFC-KIDS scale (Preckel & Strobel, 2017) assesses NFC in primary school-aged children. It consists of 14 items (e.g., “I enjoy thinking”) answered on a 3-point smiley scale (1 = *sad smiley*, 2 = *neutral smiley*, 3 = *happy smiley*) (raw score range: 14–42). We computed the scale score by building the unweighted sum across all items. In accordance with Preckel and Strobel (2017), we imputed missing values with the mean (rounded to an integer) of the non-missing items.

#### Software

We ran all analyses using *R* (4.2.1; R Core Team, 2020), and the packages *foreign* (0.8–82; R Core Team, 2022), *tidyverse* (1.3.2; Wickham et al., 2019), *psych* (2.2.5; Revelle, 2022), *cNORM* (3.0.1; A. Lenhard et al., 2018b), *gamlss* (5.4–1; Rigby & Stasinopoulos, 2005), *gamlss.tr* (5.1–7; Stasinopoulos & Rigby, 2007), *lme4* (1.1–31; Bates et al., 2015), *janitor* (2.1.0; Firke, 2021), *flextable* (0.8.5; Gohel & Skintzos, 2023), and *ggplot2* (3.4.0; Wickham, 2016). All code is available as Supplemental material (ESM 5–6).

#### Norm Score Calculation

We computed conventional, semi-parametric, and parametric age norms. We used percentile scores for all methods. For conventional and semi-parametric norms, we divided the age range (71–146 months) into subgroups. For modeling, we used 15 subgroups spanning 5 months. For the robustness check, which we describe more thoroughly later, we used seven subgroups spanning roughly 10 months. We report subsample size and statistics for the NFC-KIDS as Supplemental materials (ESM 7). For each subgroup, we conducted rank-based inverse normal transformation of the NFC-KIDS scores resulting in conventional norm scores. These scores served as input for semi-parametric norms.

##### Semi-parametric Norms

We followed the tutorial by Gary et al. (2021). We set our maximum power to five (a maximum power of four is usually sufficient to reach a desired adjusted *R*^2^ > .99; Gary et al., 2021; A. Lenhard et al., 2019; W. Lenhard & Lenhard, 2021). We compared all models with an adjusted *R*^2^ > .99 regarding additional fit criteria, namely parsimony and the root mean square error (RMSE; that is, the precision of norms across cross-validation samples), to identify the best-fitting model. Afterward, we visually evaluated the absolute fit of this model (Gary et al., 2021). Specifically, we used plots showing the percentile curves with respect to age, the first-order derivative of the resulting regression function with respect to the conventional norm score, and the deviation of the manifest and fitted data for each age group. An acceptable absolute fit allows the computation of norm scores using the resulting Taylor-Polynomial. Otherwise, model adaptions are necessary.

##### Parametric Norms

We followed the tutorial by Timmerman et al. (2021). Respecting our restricted raw score range (14–42), and the reported skewed raw score data (Preckel & Strobel, 2017) we chose a BCPE, its right truncated version, and a BB as possible PDFs. We used power polynomials and P-splines as smooth functions. Note that we only model splines for parameters where at least quadratic terms were preferred. We used the free-order procedure of Voncken et al. (2019b) to select the optimal polynomials for the four model parameters of both BCPEs. We modeled all BB-models with a maximum power of four for the two respective model parameters.

Model comparison and fit evaluation rely on the Akaike information criterion (AIC), Generalized Akaike Information Criterion with penalization factor 3 (GAIC(3)), and Bayesian information criterion (BIC) (Akaike, 1974, 1983; Burnham & Anderson, 2004). We focused on the BIC as the main criterion because it favors parsimonious models to avoid overfitting (Timmerman et al., 2021; Voncken et al., 2021a). In the next step, we visually evaluated the absolute fit of the best-fitting model using worm plots and percentile plots (Timmerman et al., 2021; van Buuren & Fredriks, 2001). Worm plots are detrended QQ-plots showing the deviation between the fitted and the theoretical distribution. Norms can be computed if there is no global and local misfit.

#### Norm Precision

We used the interpercentile range (IPR; Oosterhuis et al., 2016) as a proxy for precision. A smaller IPR indicates a smaller sample error and, thus, a higher precision. While Oosterhuis et al. (2016) estimated the IPR over simulated samples, we draw 1,000 bootstrap samples with *N* = 2,581 from the analysis sample to assess the proneness to sample fluctuation. For all samples, we computed conventional and continuous norms using the resulting models from above. We estimated the respective raw scores associated with the following percentiles: 1, 5, 10, 25, 50, 75, 90, 95, and 99. Unlike Oosterhuis et al. (2016), we decided to include the percentiles below the median because we did not assume a normal distribution (Preckel & Strobel, 2017). By having the raw scores associated with these percentiles, it is possible to calculate IPRs with respect to age group and percentile.

We conducted this analysis twice to increase the robustness of our results. First, with the number of subgroups used for modeling (15) and second with a reduced number of subgroups (7), which results in an increased sample size per subgroup. We considered the varying sample size in the bootstrap samples in our IPR calculation. Overall, these analyses result in 9 (percentiles) × 15 (seven; age subgroups) IPRs. We summarize this set of IPRs by extracting the maximum and mean values.

#### Norm Tables

To investigate whether building discretized norm tables for continuous norms introduces transition or within-subgroup bias, we considered different scenarios with varying numbers of groups. We examined scenarios with two to 75 groups. We compared the transition and within-subgroup bias of continuous norms and conventional norms. Due to precision concerns, we set a minimum of *n* = 20 per age subgroup for all analyses using conventional norms. Thus, we only built a maximum of eight subgroups for conventional norms. Consequently, we conducted the following analyses with conventional norm scores of our participants as well as for continuous norms using the mean age of each age subgroup as norm predictor. We evaluated the transition and within-subgroup bias of discretized norm tables for the different scenarios. We also estimated the differences between individual conventional and continuous norm scores for the different scenarios.

We assessed transition bias and within-subgroup bias for the scenarios described. Transition bias is prevalent when the norm score assigned to the same raw score differs substantially between two neighbored subgroups. Thus, we assess transition bias as the discrepancies in norm scores assigned to the same raw score of two neighboring age subgroups (e.g., Groups 2 and 3). For example, a raw score of 30 in subgroup two is associated with a percentile of 34, while in Subgroup 3 it may be associated with a percentile of 38. We estimate this discrepancy for all possible raw scores and age subgroups. This estimate provides a generalized indicator for the gray arrow in Figure 1, which only shows discrepancies at the median. Overall, this results in the number of groups minus one (number of possible transitions) × 29 (possible raw scores) differences. As an outcome, we report the maximum absolute transition bias for each scenario.

Within-subgroup bias is prevalent when there is a correlation between norm predictor (i.e., age) and norm score within a subgroup. However, simply estimating the correlation between norm predictor and norm score within each subgroup is not sufficient as an indicator of within-subgroup bias. The correlation depends on the sample size of the subgroup as well as on the (restricted) variance of the norm predictor within the subgroup. As an alternative but conceptually similar estimate, we used the slope within each subgroup as an indicator of the within-subgroup bias. To estimate this slope, we need to compare the norm score assigned to a specific raw score at the lower margin with the norm score assigned to the same raw score at the upper margin. For the lower margin, we used the norm score assigned to the same raw score in the neighboring lower subgroup. For the upper margin, we used the norm score assigned to the same raw score in the neighboring higher subgroup. As example, to estimate the norm score assigned to a specific raw score at the lower margin of Subgroup 3, we used the norm score assigned to the same raw score in Subgroup 2. Similarly, for the upper margin of Subgroup 3, we used the norm score assigned to the same raw score in Subgroup 4. The discrepancy between the norm scores assigned to a specific raw score at the lower and upper margin is a proxy for the slope and, thus, the within-subgroup bias. The blue arrow in Figure 1 illustrates this proxy. Note that this proxy tends to overestimate the within-subgroup bias and therefore forms an upper boundary. Again, we took the maximum within-subgroup bias for each scenario as the outcome.

#### Norm Discrepancies

To examine the change in individual norm scores across different norming methods, we calculated the differences between individual conventional and continuous norm scores. We conducted this analysis for all possible scenarios. Contrary to Thompson et al. (2022), we used the absolute differences as the outcome to avoid compensating effects of negative and positive differences. We investigated the trend of mean and maximum differences across the possible scenarios descriptively.

#### Remaining Relation With Age

We examined whether the norming methods could reduce the linear relationship between norm scores and age. As the data structure was nested (i.e., students within classes), we used the standardized effects of multilevel models as correlation index. NFC norm scores were the criterion variable, age was the predictor variable, and classroom was the group variable. Based on the ΔICC of stepwise tested intercept-only, random-intercept-fixed-slope, and random-intercept-random-slope models, we used a random-intercept-fixed-slope model as the final model. We report these models as Supplemental material (ESM 11). Again, we conducted these analyses across all possible scenarios.

### Results

#### Descriptive Statistics

The NFC-KIDS scale had a mean value of *M* = 33.45 (*SD* = 5.46). Reporting this mean in a standardized form, namely the *Percentage of Maximum Possible* (*POMP* = 69.46), revealed an easy difficulty of the scale. The negative skewness (ν = −0.52) supports this assumption. Nonetheless, we detected no severe violations of normality considering the small excess (γ = 0.08) and the reported skewness (West et al., 1995). Reliability analysis revealed a good internal consistency of the scale (ω = .86). We report age subgroup descriptive statistics and reliability as supplemental material (ESM 7). Bivariate correlations of NFC-KIDS with age and age subgroup were both *r* = −.17 (*p* < .001) indicating that younger children reported higher levels of NFC.

#### Semi-Parametric Norms

The models having at least a maximum power of two reached an adjusted *R*^2^ > .99. For parsimony reasons, we excluded a model with a maximum power of five. Of the remaining models, the one with a maximum power of four achieved the lowest *RMSE* of 0.60. It consisted of five terms in addition to the intercept. In the following regression functions, *r* refers to the test raw score, *l* to the conventional norm score, and *a* to age:

$$r\left(l,a\right)=2.15+0.72l-3.48E^{-7}l^{4}-0.07a^{2}+3.63E^{-3}a^{3}+1.69E^{-9}l^{4}a,$$

**Figure 4.**
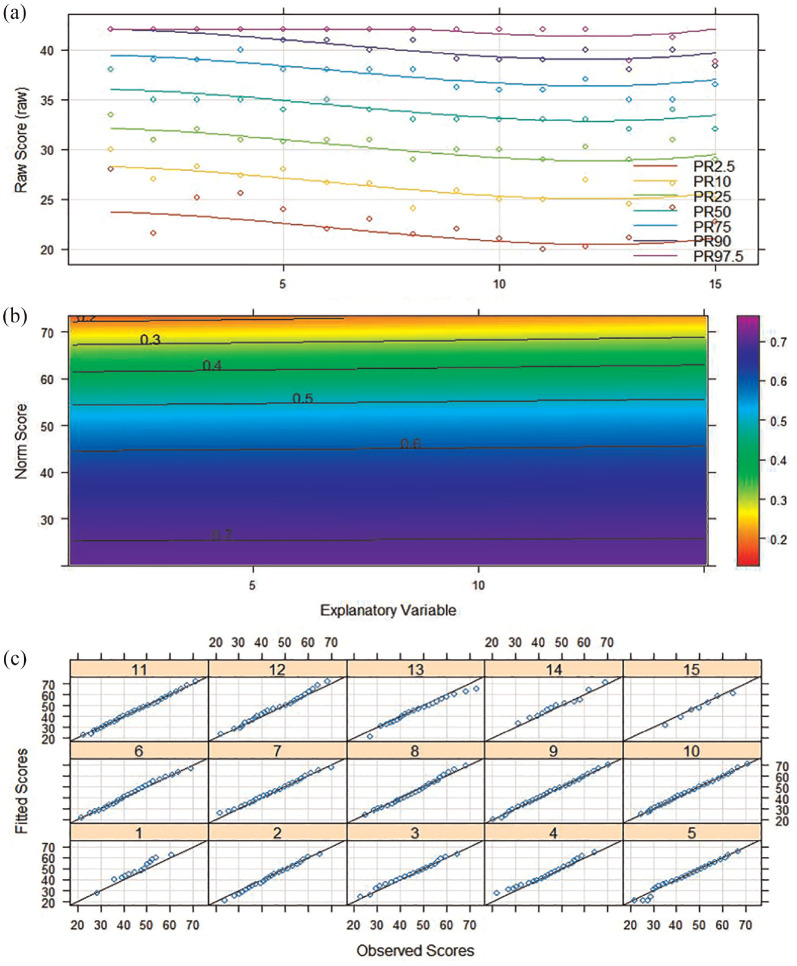
Percentile Plot (a), First-Order Derivative of Norming Regression Function (b), and Deviation Plot Between Observed and Fitted Norm Scores for the Different Age Groups (c) for Semi-Parametric Norming Model. *Note*. Model-implied percentiles (lines) align with observed percentiles (dots) in the percentile plot and shows no intersections (a). The first*-*order derivative of the norming regression function does not indicate zero-crossings (b). The deviation plot shows pp-plots for the modeled age subgroups, where only small deviations between model-implied and observed percentiles occur (c). Combined, this implies a good absolute fit.

Figure 4 shows the percentile plot, the first-order derivative of the resulting regression function with respect to the conventional norm score, and the deviation of the manifest and fitted data for each age group. The absolute fit was acceptable. The percentile plot shows no intersections of the model-implied percentile lines. This finding is supported by the absence of zero-crossings of the first-order derivative. Finally, there are only small deviations between the observed and fitted norm scores.

#### Parametric Norms

Table 3 shows the relative fit indices of the best-fitting models combining the three PDFs and two smooth functions. We encountered convergence issues for higher than quadratic terms for the kurtosis parameter for the BCPE. Therefore, we manually modeled this function based on the first and unproblematic iterations of the free-order procedure (Voncken et al., 2019b). Moreover, splines seemed reasonable for this parameter. Quadratic terms were not preferred for any other parameter or function, but we report the spline models for completeness. We report the fit statistics of all final and manually computed models as Supplemental material (ESM 8).

**Table 3:** Fit Indices of the Final Models

Probability density function	Smooth function	AIC	BIC	GAIC(3)
BCPE(1,0,1,1)	Power Polynomials	15,779	15,820	15,786
BCPE(1,0,1, S)	Penalized Splines	15,830	15,859	15,835
BCPEtr(1,0,0,0)	Power Polynomials	16,016	16,034	16,019
BCPEtr(S,0,0,0)	Penalized Splines	16,089	16,101	16,091
**BB(1,1)**	**Power Polynomials**	**15,713**	**15,736**	**15,717**
BB(S, S)	Penalized Splines	15,775	15,792	15,778

*Note*. Values in parentheses behind the respective probability density function represent the power of each parameter (i.e., μ, σ, ν, and τ for BCPE(tr) models and μ and σ for BB). S indicates Splines for this parameter. Final model is displayed in bold. AIC = Akaike Information Criterion. BIC = Bayesian information criterion. GAIC(3) = Generalized Akaike Information Criterion with penalization factor 3.

**Figure 5.**
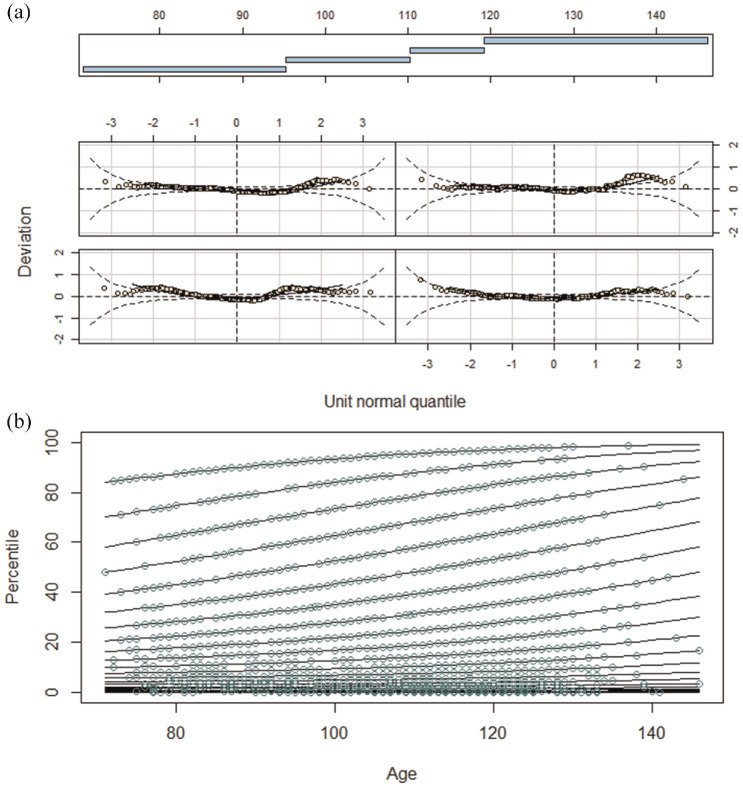
Worm Plot (a) and Percentile Plot (b) of BB-Model of Parametric Norming. *Note*. The blue bars above the worm plots (a) indicate the age range for each of the four plots arranged in rows. The youngest age quantile is represented in the bottom left plot (1), while the eldest is shown in the top right plot (4). The worm plots are flat and generally fall between the two semi-circles, indicating a good absolute fit. In the percentile plot (b), dots represent observed percentiles dependent on age, and each line corresponds to a raw score indicating the model-implied percentile with respect to age. The plot demonstrates a monotonically increasing trend, suggesting a low likelihood of overfitting.

Overall, the model with the BB function and power polynomials exhibited the best relative fit. Figure 5 displays the absolute fit visually. The worm plots appeared mostly flat, indicating only small deviations between the fitted and the theoretical distribution. The percentile plot showed a smooth trend, following the negative correlation of age and NFC. We did not assume overfit since the final model contained only linear terms for both distribution parameters. This is also evident from the monotonic trend of the percentile curves. Given the absence of local or global misfit, this model was accepted for norm score computation.

#### Norm Precision

Table 4 presents the average and maximum IPRs categorized by norming method and number of age groups. We attached tables showing the specific IPRs for each percentile, age group, and method as Supplemental material (ESM 9). Evaluation of the IPRs revealed a clear hierarchy of norming methods, with conventional norms being the least precise, followed by semi-parametric norms, and parametric norms being the most precise. Semi-parametric norms were 2 to 3 times more precise than conventional norms, while parametric norms were 4 to 6 times more precise than conventional norms.

**Table 4:** Average and Maximum IPRs for the Different Norming Methods and Number of Groups

Norming method	Number of age groups	Average IPR	Max IPR
Conventional	15	3.02	11
Conventional	7	1.92	10
Semi-parametric	15	1.17	6
Semi-parametric	7	1.02	4
Parametric	15	0.50	2
Parametric	7	0.44	2

*Note.* Higher IPRs indicate a higher sampling error and a lower precision.

Descriptively, there was an interaction between norming method and number of groups. While the number of groups had a strong influence on the precision of conventional norms, its effect dwindled for the continuous norming methods. The impact on conventional norms was a consequence of the negative correlation between sample size per subgroup and number of groups. Thus, the smaller the sample size per age subgroup the lower the precision of conventional norms.

#### Norm Table Bias

**Figure 6.**
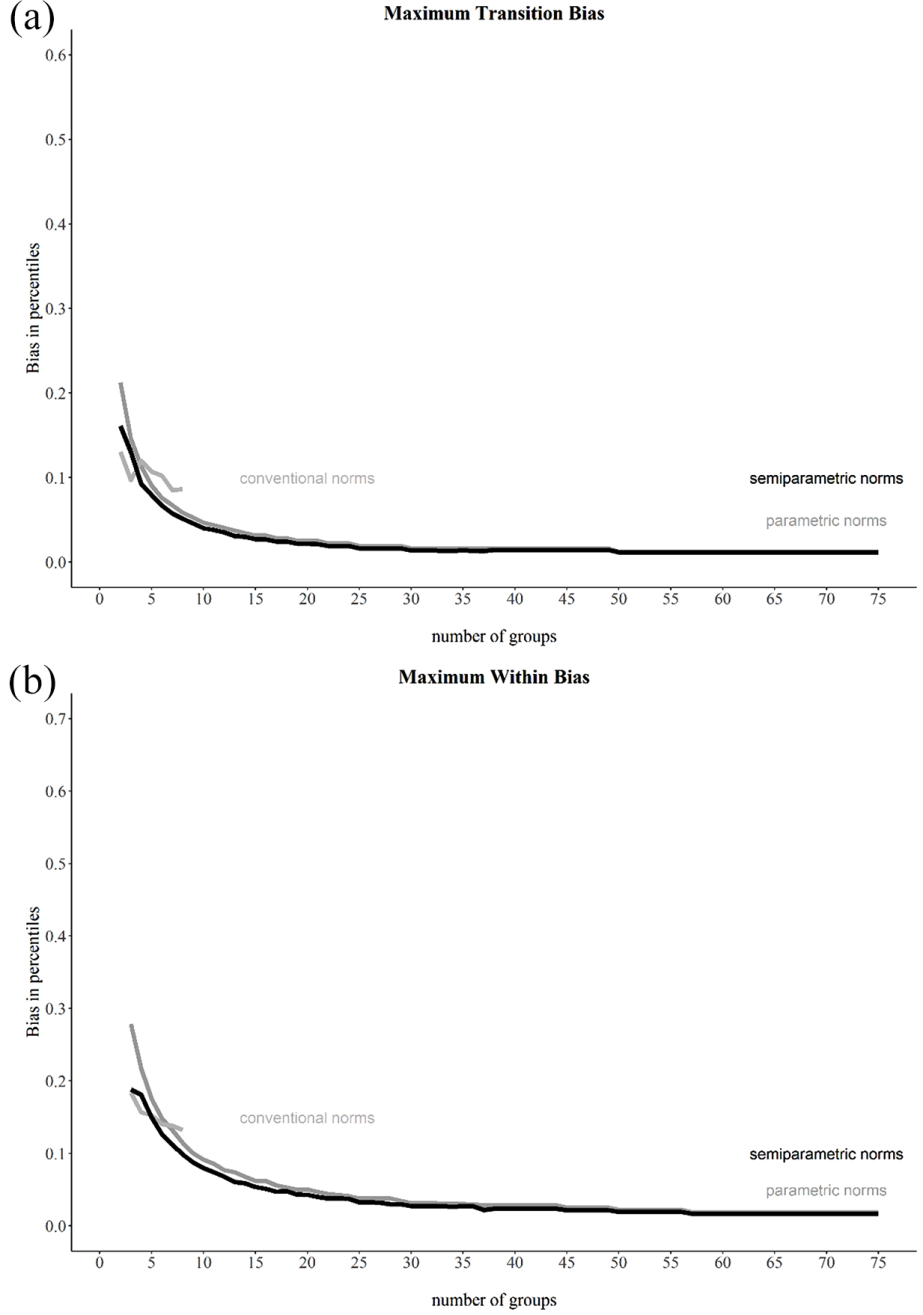
Maximum Values of Transition Bias (a) and Within-Subgroup Bias (b) for Parametric, Semi-Parametric, and Conventional Norms. *Note.* We computed bias for just eight subgroups for conventional norms by setting the minimum subgroup sample size to *n* = 20.

Figure 6 presents the maximum transition and within-subgroup bias for each scenario for the three norming methods. When only a few groups are available, the norm tables of all norming methods showed transition and within-subgroup bias. However, transition and within-subgroup bias decreased with an increasing number of groups for both continuous norming methods following an inversely proportional trend. For conventional norms, transition bias values varied around 0.10 and within-subgroup bias varied around 0.15. Both continuous norms achieved lower values for transition and within-subgroup bias than conventional norms when at least four (transition bias) or six (within-subgroup bias) norm groups were built. We attached the absolute maximum transition and within-subgroup bias values as Supplemental material (ESM 10).

#### Norm Discrepancies

**Figure 7.**
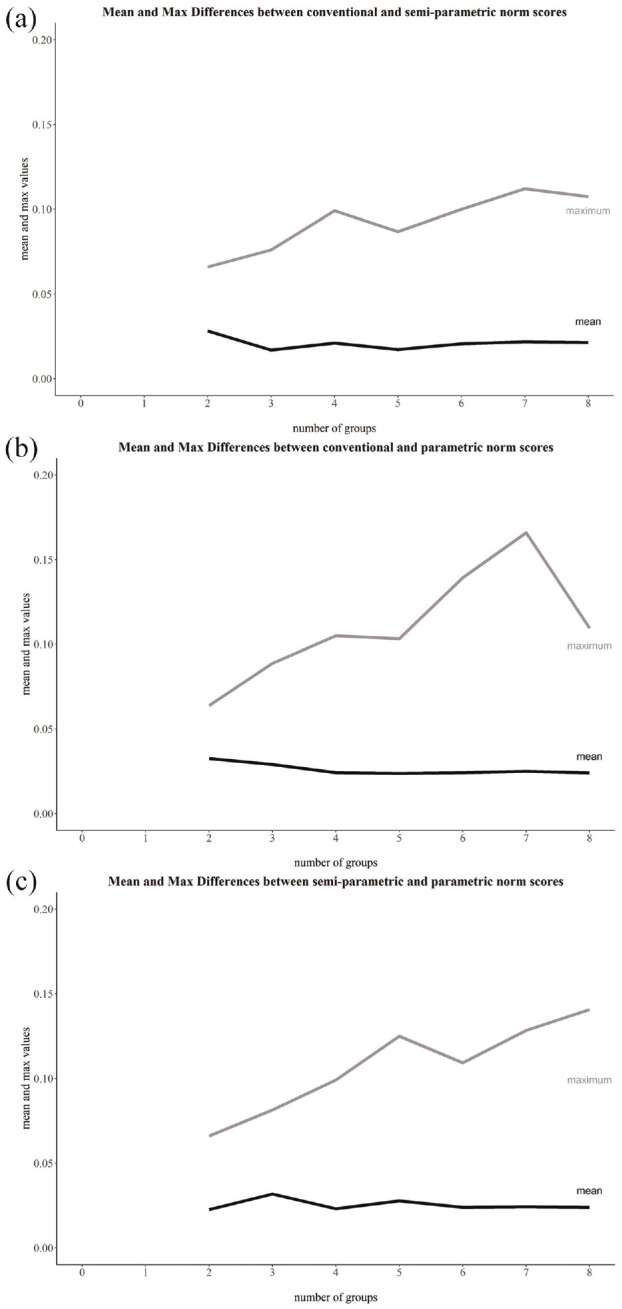
Mean and Maximum Absolute Differences Between Individual Norm-Scores.

In general, the absolute differences between individual conventional and semi-parametric norms (*M* = 0.02, *Mdn* = 0.02, *SD* = 0.01), conventional and parametric norm (*M* = 0.03, *Mdn* = 0.02, *SD* < 0.01), and semi-parametric and parametric norms (*M* = 0.02, *Mdn* = 0.02, *SD* < 0.01) were small. The maximum difference found between an individual norm score was greatest between conventional and parametric norms (*Max* = 0.17), followed by conventional and semi-parametric norms (*Max* = 0.11), and semi-parametric and parametric norms (*Max* = 0.14). While the difference may be small for most individuals, the norming method made a large difference for some individuals (up to 17 percentiles). We plotted the relationship between the number of groups with mean and maximum differences in Figure 7.

#### Remaining Relation With Age

The remaining correlation of NFC and age varied between *r* = −.01 and *r* = .01 for conventional norms, between *r* = −.04 and *r* = .01 for semi-parametric norms, and between *r* = −.01 and *r* = .02 for parametric norms across the possible scenarios. All norming methods were equally effective in eliminating the linear effect of age.

### Summary

Our real data study with the slightly skewed norm data of the NFC-KIDS included *N* = 2,581 participants and aimed to address open questions in the continuous norming literature. We compared the precision of conventional, semi-parametric, and parametric norms. We assessed whether discretized norm tables introduced transition and within-subgroup bias for conventional and continuous norms. We investigated differences between individual conventional and continuous norm scores. Finally, we assessed the ability of the resulting norms scores to reduce the linear effect of age on NFC.

We observed a clear hierarchy of the three norming methods examined. Precision increased from conventional to semi-parametric to parametric norms. Moreover, the number of subsamples and consequently the subsample size primarily affected conventional norms. Using only a few groups to build discretized norm tables introduced transition and within-subgroup bias for all norming methods. Transition and within-subgroup bias decreased as the number of groups increased. Absolute mean differences between individual norm scores were negligible. Nonetheless, the maximum differences were substantial. Despite differences in precision, conventional and continuous norms reduced the linear effect of age on NFC. However, any age-subgroup specific transformation of NFC raw scores would have reduced the systematic (linear) relationship between age and NFC. Thus, the analysis of the remaining correlation (Brickenkamp et al., 2010) cannot be taken as an indicator that the aim of norming–expressing a test raw score in a standardized way–has been achieved.

## General Discussion

In this study, we aimed to provide a comprehensive review of continuous norming and to address research gaps identified in that review. We conducted a systematic review of journal articles and augmented it with an empirical study using the NFC-KIDS norm data. The main findings of our review indicate that most studies used simplified parametric norming, that not all studies considered essential distributional assumptions, and that the evidence comparing different norming methods is inconclusive. In our real data example, a hierarchy in terms of norm precision emerged with conventional norms being least precise, followed by semi-parametric norms, and parametric norms being most precise.

### Precision and Bias

Our findings that continuous norms suffer less from sampling fluctuations and are thus more precise than conventional norms align with previous studies (e.g., Crompvoets et al., 2021; Oosterhuis et al., 2016). Moreover, in our robustness check, we found that the number of age subgroups only affected the precision of conventional norms. Continuous norms remained consistently precise, and the precision hierarchy did not change. This invariance of continuous norms and the precision hierarchy is fully consistent with previous studies (Eid & Schmidt, 2014, p. 381; A. Lenhard et al., 2018a; Oosterhuis et al., 2016) and with the fact, that the subgroups are irrelevant for parametric norm computation (Timmerman et al., 2021).

Contrary to earlier studies comparing semi-parametric and parametric norms (Gary & Lenhard, 2021; A. Lenhard et al., 2019), we found a precision advantage for parametric norms. Moreover, this advantage persisted in our skewed data. Differences in skewness and modeling may account for these different results. Our final model used a BB as the PDF with power polynomials as smooth function. Neither was used in previous comparative studies (Gary & Lenhard, 2021; A. Lenhard et al., 2019). Furthermore, we had a larger sample size than most studies comparing different norming methods. Earlier studies have shown that sample size affects the absolute precision but that it does not affect which norming method provides more precise norms (Crompvoets et al., 2021; Gary & Lenhard, 2021; A. Lenhard et al., 2019). Moreover, our finding that continuous norms are 2 to 6 times more precise than conventional norms aligns with prior research (Oosterhuis et al., 2016). We approximatively replicated IPR ratios between conventional and continuous norms. Oosterhuis et al. (2016) found that continuous norms were 3 to 5 times more precise than conventional norms. Note that Oosterhuis et al. (2016) used simplified parametric norming, which limits the exact comparison of the IPR ratios.

A high precision of norms does, however, not guarantee the absence of bias (e.g., Crompvoets et al., 2021). As we used a real data example, we cannot ensure that our continuous norming models matches the population model. Nonetheless, we consider (a large) bias in our continuous norming models to be unlikely. We reached this conclusion for two reasons. First, we followed the respective recommendations to avoid under- and overfit (Gary et al., 2021; A. Lenhard et al., 2018a; Voncken et al., 2021a). We used the BIC to select a parametric norming model, selected a semi-parametric model with an *R*^2^ > .99, and visual fit assessment showed no evidence of overfit (Figures 4 and 5). Second, we observed that mean differences between conventional and continuous norms were mostly small. Furthermore, the pattern of differences between parametric norms and conventional norms was similar to that between semi-parametric norms and conventional norms. If any continuous norming method were biased, we would expect larger differences and a different pattern.

### Norm Table Bias and Norm Discrepancies

Even continuously normed tests present classical norm tables that show the relationship between raw scores and norm scores (e.g., Grob & Hagmann-von Arx, 2018; Kubinger & Holocher-Ertl, 2014; W. Lenhard et al., 2017). Such discretized norm tables may introduce transition and within-subgroup bias. We investigated whether these biases occur for all norming methods across a varying number of subgroups. For a small number of groups, norm tables of all norming methods showed transition and within-subgroup bias. Both biases decreased for continuous norms as the number of subgroups increased. The effect of adding one group on reducing bias decreased as the number of subgroups increased. Our analyses do not allow us to determine a minimum number of subgroups needed to ensure that discretized norm tables are free of transition and within-subgroup bias. However, we encourage test developers to use our or a similar approach to verify that their discretized norm tables of continuous norms do not introduce transition or within-subgroup bias.

In addition, we examined the effect of the number of groups on the discrepancy between individual conventional and continuous norm scores. The analysis was only possible for a maximum of eight subgroups. Substantial maximum differences occurred especially between individual conventional and parametric norm scores. This highlighted that a fine-grained norm differentiation is only appropriate if the precision is sufficient. Nonetheless, it does not answer the question of whether a fine-grained norm differentiation is necessary.

We examined the effects of norming method and number of subgroups in discretized norm tables on the linear correlation between norm scores and the norm predictor age. Neither norming method nor number of subgroups had an effect. The linear correlation was close to zero for all conditions. However, as shown in our analysis of within-subgroup bias, there was a relationship between age and norm scores as a function of norming method and number of subgroups. We conclude that a linear correlation of zero between norm predictor and norm scores does not guarantee that norm scores and the norm predictor are independent.

### Limitations

Our review has some limitations. We achieved a moderate Krippendorff’s alpha of α = .76. Interrater discrepancies led to overinclusion. Four of the five discrepant studies were included in the review. Therefore, we did not assume a lack of sensitivity or specificity.

There may be a publication type bias, as tests and norming are predominantly published as test manuals rather than as journal articles or dissertations. However, it is unclear whether publication type affects the sample characteristics and applied norming methods in general. We addressed this limitation in a systematic overview of German tests (Urban et al., in press).

Our study faces similar issues as previous research comparing different norming methods (Gary & Lenhard, 2021; A. Lenhard et al., 2019). Results could be different if we selected alternative models for continuous norming. Despite working with non-optimal real data, we were able to find a well-fitting parametric model. The generalizability of our results to other real data studies and tests is, nonetheless, restricted. Our final parametric norming model included only linear terms and the linear correlation of age and NFC raw scores was *r* = −.17. More complex terms or stronger initial associations with age may affect the possibility to find well-fitting parametric norming models. Similar to other studies (e.g., A. Lenhard et al., 2018a), we did not account for the nested data structure when estimating the continuous norming models. Whether a nested data structure affects model selection, precision, or bias in continuous norming is yet unclear and remains a topic for future research.

More complex terms can also affect the method of transition and within-subgroup bias estimation. If the percentile curves (Figure 5) are not monotonic (e.g., curvilinear associations), our within-subgroup bias estimator will underestimate the bias. We used the neighboring subgroups to define the norm score assigned to a given raw score at the margin of one age subgroup. Here, continuous norms enable the exact estimation of this association at the margin. This estimation is not possible with conventional norming. We highly recommend this exact estimation when no comparison to conventional norms is needed. To solve the issue of lacking monotonicity, we suggest splitting the age subgroups at the inflection points of percentile curves. This ensures monotonicity within each subgroup.

We used age subgroups of equal width. For example, we built 15 age subgroups spanning 5 of months each. A more detailed splitting strategy that considers inflection points of percentile curves seems preferable. Moreover, the percentile at which the maximum transition or within-subgroup bias occurs, is of interest. Both biases are more likely to occur in regions with high test score density. The individual consequences of such biases tend to be higher in regions with low test score density. Thus, considerations such as trend, percentiles, reliability, consequences of testing, and the application field of the test in general should guide the splitting into age subgroups.

Finally, the generalizability of our empirical findings is limited because we investigated one specific data set. Our data related to a large sample (*N* = 2,581), 14 items with a 3-point scale, slightly negative skewness, and primary school age children (71–146 months). We modeled a (truncated) BCPE and BB as PDF with power polynomials and P-Splines as smooth functions for parametric norming. We selected a semi-parametric model based on *R*^2^ > .99, parsimony and the RMSE. Although we cannot generalize our results to other data sets or modeling strategies, we add nuance to prior findings comparing different norming methods.

### Theoretical and Practical Implications

Model selection plays a crucial role in continuous norming, as highlighted in our study. Modeling precise parametric norms is possible even with skewed data. The selected PDFs and smooth functions affect precision and bias, emphasizing the importance of comprehensive testing. In the absence of a comprehensive testing and fit evaluation (simplified) parametric norms may misfit the data and consequently show a lower precision or higher bias than other norming methods (e.g., Crompvoets et al., 2021; Gary & Lenhard, 2021; A. Lenhard et al., 2019). To avoid misfit and to balance over- and underfitting, we recommend following the guidelines of Gary et al. (2021) for semi-parametric norming and using the BIC for parametric norming (Timmerman et al., 2021; Voncken et al., 2019b). Moreover, it is essential to respect the model assumptions specific to the chosen norming method or model.

Continuous norming is beneficial if implemented properly. As a tutorial, we recommend the guidelines of Gary et al. (2021) and Timmerman et al. (2021). When comparing the different continuous norming methods, inferential norming and simplified parametric norming seem less complex. However, inferential and simplified parametric norming can lead to biased norms if model assumptions are not tested or respected (e.g., Crompvoets et al., 2021; Innocenti et al., 2023; Oosterhuis et al., 2016). Model assumptions are also relevant to parametric norming. Moreover, parametric norming itself is more sophisticated than semi-parametric norming, especially when model selection is conducted thoroughly. Although we have found evidence of an advantage for parametric norms, it is necessary to assess the return on investment—does the greater effort of thorough parametric model selection pay off in terms of precision and bias compared with semi-parametric norming? To evaluate this return on investment, we encourage the reporting of the norming procedure and model fit. Both are often reported briefly, despite their importance for appropriate modeling.

A second practical implication concerns the reporting of discretized norm tables for continuously normed tests. Building too few groups can introduce transition and within-subgroup bias. We encourage test developers to consider whether their discretized norm tables introduce transition or within-subgroup bias. To do so, test developers can use an approach similar to the one presented in our real data example, by estimating transition and within-subgroup bias using different number of groups. The final decision on the number of subgroups should also consider interpretability, comprehensibility, the number of tables, and theoretical considerations, such as the true construct change, to avoid unnecessarily narrow subgroups.

Our final practical implication relates to the sample size required to compute precise continuous norms. Previous studies mainly focused on inferential norming or simplified parametric norming (Innocenti et al., 2023; Oosterhuis et al., 2016; Zhu & Chen, 2011). However, the guidelines of Oosterhuis et al. (2016) may also be applicable for semi-parametric and parametric norms since we approximately replicated the IPR ratios between conventional and continuous norms of Oosterhuis et al. (2016). Since the study of Oosterhuis et al. (2016) differed from ours in terms of number of items and number of response points such recommendations need validation. Moreover, the combination of overall sample size and the total range of the norm predictor are also relevant for continuous norming.

We recommend oversampling participants at the edges of the norm predictor range and at inflection points of the norm predictor-raw score association. The rationale for this is that neighboring subgroups will compensate for random sample error within a subgroup, which is not the case for groups at the edges and groups with inflection points. Alternatively, Timmerman et al. (2021) suggested sampling participants with a norm predictor value slightly outside the norm predictor range of the target population, provided that the test is appropriate for individuals with a norm predictor outside the range.

### Future Research Directions

The question of whether semi-parametric or parametric norms are preferable remains unanswered. It is a desiderate to evaluate the influence of different factors on these methods in simulation studies and to validate the results in real data studies. The different factors should be, according to Gary and Lenhard (2021), A. Lenhard et al. (2019), Oosterhuis et al. (2016), and Timmerman et al. (2021), the scale difficulty, number of items, response scale, the effect of covariates, norm predictor range, and sample size. An additional factor that received little attention in continuous norming research is reliability (W. Lenhard & Lenhard, 2021; Voncken et al., 2019a). Lower reliability, and thus more random variance, may particularly affect the trade-off between precision and bias (Voncken et al., 2021a). The outcome variables of such simulation studies are the resulting norming model, precision, bias, and discrepancies between the individual norm scores. Moreover, such studies can provide further recommendations for the required sample size, which is an important practical implication for test developers.

Sample size may be important not only for precision of norms, but also for correct model selection. To our knowledge, there are no studies on the sensitivity of applied model selection procedures to identify the correct population model. For example, a BCPE with quadratic effects for each distribution parameter describes the association of raw score and norm predictor in the population. How sensitive is the free-order procedure (Voncken et al., 2019b) in identifying these parameters? Simulation studies with different population models, different raw score norm predictor associations, and different sample sizes are desirable to test the sensitivity of model selection. Determining sensitivity seems important because misspecifications can lead to highly precise, but systematically biased, norm scores.

Another open question concerns applied research that uses norm scores rather than raw scores. In such studies, researchers examine associations between different variables assessed via test or questionnaires frequently using norm scores of the respective measures. Of these norm scores, researchers typically compare group means or investigate (co)variances of norm scores (e.g., Breit et al., 2022; Hoekstra et al., 2007; Liew et al., 2018). With respect to means, we found no large differences between the norming methods, which is in line with Thompson et al. (2022), but the results lack generalizability. To evaluate the robustness of means and (co-)variances across norming methods, further simulation studies are needed. The results of these studies should be validated by replicating existing journal articles with the originally used conventional norms and newly computed continuous norms.

## Conclusion

Our review showed a growing interest in continuous norming. Most studies used simplified parametric norms, with fewer studies using inferential, semi-parametric, or parametric norming. Despite this growing interest, open questions remain. For example, under what circumstances is which norming method preferable, or what sample size is required? In our real data study, we added to our knowledge of these open questions. We found that continuous norming methods, especially semi-parametric and parametric norming, provided precise norms. Thus, they represent a possible solution to the economy-precision dilemma for continuous norm predictors. These advantages are of immense relevance given the potential impact of biased and imprecise norms on individuals. To advance knowledge about continuous norms, we recommend that test developers and researchers use and study semi-parametric and parametric norms.
